# Separating Surface Reflectance from Volume Reflectance in Medical Hyperspectral Imaging

**DOI:** 10.3390/diagnostics14161812

**Published:** 2024-08-20

**Authors:** Lynn-Jade S. Jong, Anouk L. Post, Freija Geldof, Behdad Dashtbozorg, Theo J. M. Ruers, Henricus J. C. M. Sterenborg

**Affiliations:** 1 Department of Surgery, Netherlands Cancer Institute, Plesmanlaan 121, 1066 CX Amsterdam, The Netherlands; 2 Department of Nanobiophysics, Faculty of Science and Technology, University of Twente, Drienerlolaan 5, 7522 NB Enschede, The Netherlands

**Keywords:** hyperspectral imaging, glare, specular reflection, optical phantoms, breast tissue

## Abstract

Hyperspectral imaging has shown great promise for diagnostic applications, particularly in cancer surgery. However, non-bulk tissue-related spectral variations complicate the data analysis. Common techniques, such as standard normal variate normalization, often lead to a loss of amplitude and scattering information. This study investigates a novel approach to address these spectral variations in hyperspectral images of optical phantoms and excised human breast tissue. Our method separates surface and volume reflectance, hypothesizing that spectral variability arises from significant variations in surface reflectance across pixels. An illumination setup was developed to measure samples with a hyperspectral camera from different axial positions but with identical zenith angles. This configuration, combined with a novel data analysis approach, allows for the estimation and separation of surface reflectance for each direction and volume reflectance across all directions. Validated with optical phantoms, our method achieved an 83% reduction in spectral variability. Its functionality was further demonstrated in excised human breast tissue. Our method effectively addresses variations caused by surface reflectance or glare while conserving surface reflectance information, which may enhance sample analysis and evaluation. It benefits samples with unknown refractive index spectra and can be easily adapted and applied across a wide range of fields where hyperspectral imaging is used.

## 1. Introduction

Hyperspectral imaging is an optical technique that combines conventional imaging with spectroscopy to obtain both spatial and spectral information of a sample [1,2,3]. To obtain a hyperspectral image, a sample is illuminated with a broadband light source covering the visible and/or near-infrared wavelength range, and the reflected light is detected by a camera. Over the years hyperspectral imaging has been used in a wide range of fields, such as medicine [4], remote sensing [5,6], agriculture [7,8], food safety inspection and control [9,10,11], forensics [2,12], archaeology [13,14], surveillance [15,16], environmental monitoring [17,18] and biotechnology [19].

Hyperspectral imaging has the advantage of being a harmless, non-contact, and non-invasive technique that does not require the administration of contrast agents. It enables fast data acquisition so that necessary information can be obtained, analyzed, and monitored quickly. These factors make it particularly useful for medical diagnostics, e.g., for blood vessel detection and perfusion evaluation [20,21,22,23], oxygen saturation assessment [24,25,26] and assessment of resection margins during cancer surgery [27,28,29,30,31,32]. The spectra in the hyperspectral image are influenced by the composition and morphology of a sample. Therefore, differences in measured spectra are used to determine the state of the sample—for example, whether fruit is ripe or unripe, or whether tissue is healthy or cancerous.

**Figure 1:**
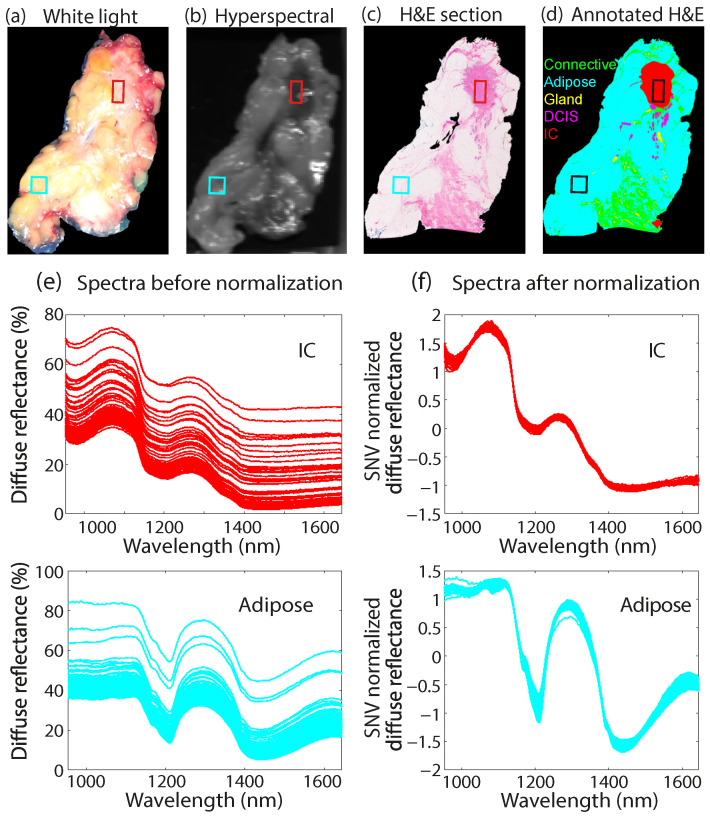
Intensity variations between measured spectra in resected breast tissue. (**a**) White light and (**b**) hyperspectral image of the resected breast tissue captured at a wavelength of 1271 nm, compared with (**c**) corresponding H&E stained section and (**d**) histopathology annotations of different tissue types. The red and blue regions (squares) represent invasive carcinoma (IC) and adipose tissue, respectively. The spectra corresponding to these regions are shown (**e**) before and (**f**) after SNV normalization. Figure adopted and modified from Kho et al. [27].

In a previous study by Kho et al. [27], the potential of hyperspectral imaging for margin assessment in breast cancer surgery was investigated. During this study, the primary objective was to differentiate between healthy and tumor tissue within excised breast samples. A comparison was made between images captured by a regular color camera, a hyperspectral camera, and annotated hematoxylin and eosin (H&E) stained sections from histopathology, as illustrated in Figure 1a–d. The authors concentrated on various regions of the breast tissue, each comprising a different tissue type, where regions delineated by squares represent examples of invasive carcinoma (red) and adipose tissue (blue). The corresponding spectra for these regions are presented in Figure 1e. While observing a consistent spectral shape for each tissue type, they also observed a strong variable baseline offset in the spectra. To address this, the authors implemented standard normal variate (SNV) normalization, a commonly employed preprocessing technique in hyperspectral imaging studies [27,30,33,34,35,36,37,38,39]. Following SNV normalization, the baseline offset effectively disappeared from the spectra (Figure 1f). Nevertheless, this normalization procedure is also known to induce a loss of amplitude and scattering information [33].

In medical hyperspectral imaging, photons are reflected from a tissue sample through two distinct mechanisms:

1. The refractive index difference between sample and air causes specular reflectance at the tissue–air interface, i.e., reflectance from the surface.
2. Light entering the tissue is scattered randomly multiple times. Although most of the scattered light will be absorbed, a fraction manages to escape from the surface. This fraction carries distinct spectral signatures and has been inside of the tissue, i.e., reflectance from the volume.

**Figure 2:**
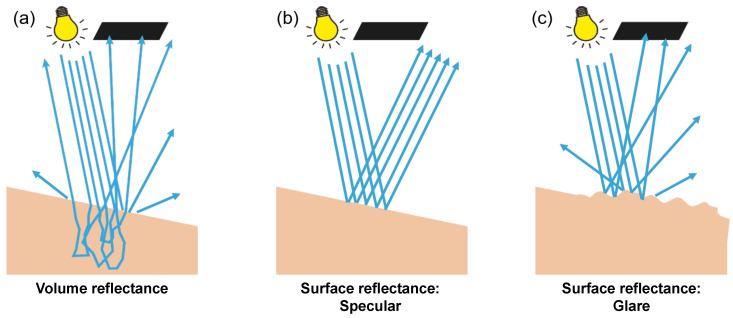
Different types of reflections. The black square indicates a pixel on the camera. (**a**) Volume reflectance of a smooth sample. (**b**) Surface reflectance of a smooth sample. The surface is placed at an angle to deflect surface reflections away from the detector. (**c**) Surface reflectance of a sample with a rough surface. Due to the presence of small surface irregularities, surface reflections can no longer be deflected away from the detector effectively. Consequently, a fraction of the surface reflections will be detected. Surface reflectance resulting from surface roughness is also referred to as glare or gloss. It exhibits a diffuse character, even though the light has never been inside the sample.

The volume reflectance is interesting for investigating bulk materials as it carries the signatures of absorption. Originating from the distribution of diffuse light inside the tissue, it exits the tissue in a broad range of angles, as depicted in Figure 2a. For this reason, volume reflectance is often referred to as diffuse reflectance. A diffuse light distribution inside the tissue has spatial gradients that are related to the scattering mean free path. Consequently, the spatial distribution of the volume reflectance on the surface also exhibits gradients related to the scattering mean free path. In other words, we see light coming out of the sample, but we cannot see very sharp structures inside.

Surface reflectance from a smooth surface follows Snell’s law as it reflects off the tissue–air interface. It is often described as specular reflectance (or a mirror-like reflectance) and exhibits a very distinct angular distribution compared to volume reflectance (Figure 2b). However, the angle of reflectance also depends on the orientation of the surface plane. When a sample has a rough surface, surface reflections occur at multiple angles and can obtain a diffuse character. This effect is illustrated in Figure 2c and is commonly known as glare or gloss.

At sufficiently large magnification, the pixel-to-pixel variations in volume reflectance are anticipated to be low because of the diffuse nature of the scattered light inside the sample. However, the observation made by Kho et al. revealed large pixel-to-pixel intensity variations. We hypothesize that this large pixel-to-pixel intensity variation is related to pixel-to-pixel variations in surface reflectance. This hypothesis comes from systematic observations indicating that the spectral shape of these variations is relatively flat, resembling the shape of specular reflectance spectra derived from the refractive indices of common tissue constituents (e.g., water, fat, etc.).

This paper investigates this hypothesis by comparing different reflectance spectra from the same pixel illuminated from three different axial angles (but the same zenith angle). A rough surface with a fine structure will cause the three contributions of the surface reflectance to differ while the volume reflectance remains the same. If our hypothesis is correct, this enables us to employ a novel approach in which surface reflectance can be separated from volume reflectance through modifications in illumination geometry and data analysis. Optical phantoms and excised human breast tissue were used in this study for pre-clinical validation, and the presented results can serve as a basis for future clinical experiments and studies.

## 2. Materials and Methods

### 2.1. General Approach to Separate Surface and Volume Reflectance

The measured reflectance $R_{tot}$ as a function of wavelength is here hypothesized to be equal to the sum of the volume reflectance $R_{vol}$ and the surface reflectance $R_{surf}$ [40]:

(1) $$R_{tot}\left(X,Y,\lambda \right)=R_{vol}\left(x,y,\lambda \right)+R_{surf}\left(x,y,\lambda \right)$$

where *X* and *Y* refer to the pixel indices and *x* and *y* to the corresponding coordinates on the surface of the sample. For readability, we omit the dependence on the pixel location (*x*, *y*), and all parameters will refer to a single pixel and its corresponding sample surface location.

The surface reflectance $R_{surf}$(λ) is proportional to the specular reflectance $R_{spec}$(λ).

(2) $$R_{surf}\left(\lambda \right)=\varepsilon \times R_{spec}\left(\lambda \right)$$

The value of the proportionality constant ε can differ between pixels as it depends on the shape and orientation of the surface [33]. For a smooth sample surface, oriented such that all the specular reflectance reaches the detector via the tissue surface, ε equals 1. For samples with a rough surface, only a fraction ε of $R_{spec}$ will reach the detector. The specular reflectance $R_{spec}$ depends on the refractive index of the sample ($n_{sample}$) and the refractive index of the medium above the sample ($n_{medium}$) and can be described using the Fresnel equations. For normal incidence, it is expressed as:

(3) $$R_{fresnel}\left(\lambda \right)={\left(\frac{n_{sample(\lambda )}-n_{medium(\lambda )}}{n_{sample(\lambda )}+n_{medium(\lambda )}}\right)}^{2}$$

In the case of a rough surface, we are not dealing with normal incidence but a wide variety of angles. Nevertheless, we assume that the shape of $R_{spec}$(λ) remains similar to $R_{fresnel}$(λ). For a mixture of fat and water, the main constituents of tissue, this spectrum is nearly wavelength independent with a deviation in the order of 1% between 500 and 900 nm.

The volume reflectance is often described by the diffusion model where the volume reflectance is a function of the reduced scattering coefficient $\mu_{s}$’ and the absorption coefficient $\mu_{a}$ of the sample [41]. The strong wavelength dependence of tissue absorption, mainly due to blood, beta-carotene, bilirubin, water, and fat, causes the volume reflectance spectrum to exhibit multiple absorption dips, i.e., a very different spectral shape compared to specular reflectance.

### 2.2. Approach: Separating Surface and Volume Reflectance

The observation that the expected spectral shapes of volume reflectance and surface reflectance spectra are very different and that the intensity of the surface reflectance strongly depends on the shape and orientation of the surface with respect to a light source and camera position motivated us to investigate a method where we acquired different images using different axial positions of the light source. We obtained a number (i) of different images, each with nearly the same volume reflectance but with a different intensity of the surface reflectance:

(4) $$R_{tot}\left(\lambda ,i\right)=R_{vol}\left(\lambda \right)+\varepsilon_{i}\times R_{spec}\left(\lambda \right)$$

where $\varepsilon_{i}$ stands for the proportionality constant from Equation (2) with respect to illumination geometry *i*.

In case we have $m_{\lambda }$ wavelengths in our spectrum and $m_{i}$ illumination geometries, Equation (4) comprises $m_{\lambda }$ × $m_{i}$ equations with 2 × $m_{\lambda }$ + $m_{i}$ unknowns (i.e., $m_{\lambda }$ for the unknown volume reflectance, $m_{\lambda }$ for the unknown surface reflectance and $m_{i}$ for the unknown surface reflectance intensities). For $m_{\lambda }$ > 2 and $m_{i}$ > 2 these equations can be solved. For $m_{\lambda }$ = $m_{i}$ = 3, there is an exact solution. For larger values, a solution requires a least squares minimalization.

To expedite the process, we will not treat $R_{vol}\left(\lambda \right)$ and $R_{spec}\left(\lambda \right)$ as arrays of unknowns, but to reduce the number of unknown variables, we will rather model them. As described above, $R_{spec}\left(\lambda \right)$ can be considered spectrally flat:

(5) $$R_{spec}\left(\lambda \right)=1$$

This approach is accurate within 2% for any mixture of fat and water. It results in a strong reduction in the number of unknowns. In addition, we modeled the volume reflectance as a polynomial of order $m_{p}$. Due to the spectral broadness of the common absorbers in tissue and the high wavelength resolution of modern spectrometers, this offers a substantial reduction of the number of unknowns, as $m_{p}$ can be ≪ $m_{\lambda }$. This gives $m_{\lambda }$ × $m_{i}$ equations with $m_{p}$ + $m_{i}$ unknowns. All minimalizations were performed in MATLAB R2023a (MathWorks, Natick, MA, USA) using the standard nonlinear minimalization function lsqnonlin. This approach does not require a priori knowledge of tissue optics-specific absorbing components, nor does it assume a homogenous medium, as is required for diffusion theory. This makes the approach widely applicable.

### 2.3. Experimental Setup

**Figure 3:**
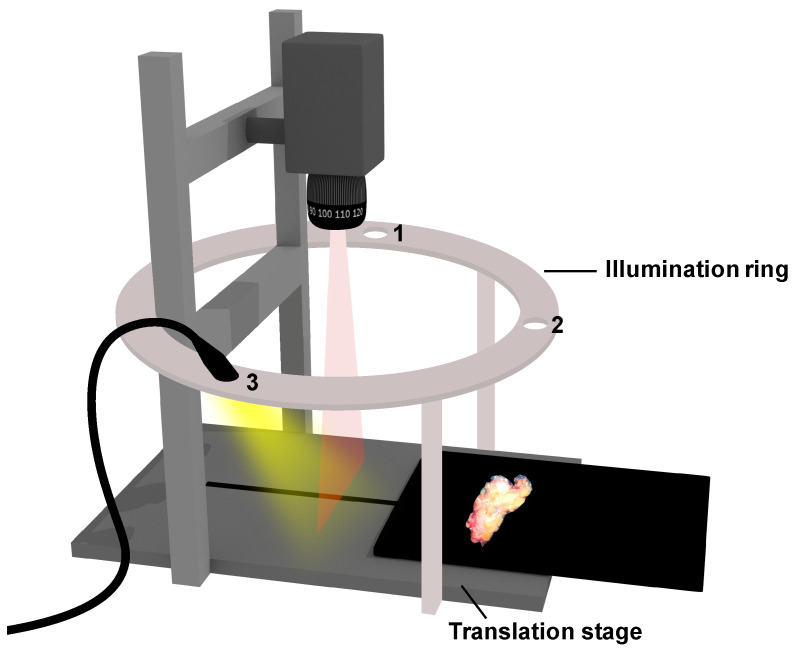
Hyperspectral imaging setup. Hyperspectral push-broom camera with a moving translation stage upon which the sample was placed and imaged line by line. The illumination ring enables a halogen light source to be mounted under a zenith angle of 30° at three different positions: 1, 2, and 3, with equal distance to the camera. By switching the position of the light source, three hypercubes of the sample can be obtained from different illumination directions (different axial directions, identical zenith angle).

To acquire the hyperspectral images, a push-broom hyperspectral imaging system (Specim, Spectral Imaging Ltd., Oulu, Finland) was used. This consisted of a camera (PFD-CL-65-V10E, linear CMOS sensor 1312 × 384), an illumination ring around the camera in which a light source (ACE, Schott Lighting and Imaging, Mainz, Germany) was mounted at three different rotational positions but under identical 30-degree zenith angles, and a motorized translation stage (LabScanner 40 × 20, Specim, Spectral Imaging Ltd., Oulu, Finland) upon which the sample could be placed. Figure 3 shows the setup. To capture an image, the sample was moved by the translation stage and imaged line by line over a wavelength range from 500 to 900 nm. This acquisition, performed with a spectral resolution of 3 nm, resulted in the generation of a 3D structure, or hypercube, with 254 wavelength bands. The acquired images had a size of 795 × 1312 pixels. The spatial resolution of 0.16 mm/pixel and the scanning speed of the translation stage were set according to the spatial resolution of the imaged line and the acquisition time of the camera. To compensate for both spectral and spatial variations in the illumination and the influence of the dark current in the setup, we performed a separate calibration for each illumination direction. We obtained a measurement with the shutter of the camera closed to determine the dark current ($I_{dark}$). Next, we measured a Spectralon reference tile (SRT-99-100, Labsphere, North Sutton, NH, USA) to obtain $I_{Spectralon}$ and then we calculated the total reflectance of the sample:

(6) $$R_{total}\left(x,y,\lambda \right)=\left(\frac{I_{sample(x,y,\lambda )}-I_{dark(x,y,\lambda )}}{I_{Spectralon(x,y,\lambda )}-I_{dark(x,y,\lambda )}}\right)\times R_{Spectralon}\left(\lambda \right)$$

where $I_{sample}$ represents the signal from the sample, and $R_{Spectralon}\left(\lambda \right)$ the specified reflectance values of the Spectralon reference tile [27].

### 2.4. Experiments

To investigate our approach, we performed measurements on two phantoms and on excised human breast tissue. To prevent reflections from below the samples, the samples were placed on a black polyoxymethylene platform, which was connected to the translation stage. Three images were acquired in sequence, each illuminated from a different position on the illumination ring but with identical 30° zenith angles as shown in Figure 3.

The data analysis was performed in MATLAB R2023a (MathWorks, Natick, MA, USA). We calibrated the hyperspectral data of the samples with respect to total reflectance as explained in Equation (6). Since the refractive index spectrum of these samples was not known, we approximated $R_{spec}$ as constant with respect to the wavelength.

For saturated pixels, this approach becomes ineffective. To identify saturated pixels, we set a threshold for the maximum value of $I_{sample}\left(\lambda ,i\right)$, equal to 4000, which is close to the maximum count number of 4096 that our 12-bit cameras could produce.

### 2.5. Phantoms

**Figure 4:**
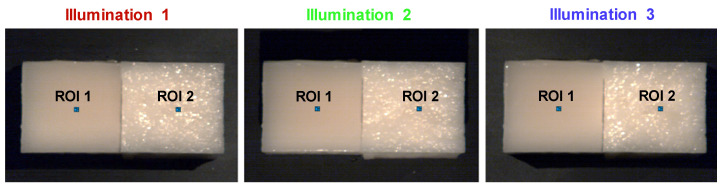
Two homogeneous optical phantoms with different surface structures imaged from three different axial angles. Pseudo-RGB images with dimensions of 550 × 300 pixels are generated from the hypercubes using wavelength bands at 900, 752, and 602 nm for red, green, and blue, respectively. The left square represents the phantom with a smooth surface, and the right square represents the phantom with a rough surface. The left phantoms appear very similar to each other, whereas those on the right vary in terms of detail. A region of 25 pixels is marked on both phantoms for each illumination direction, indicating the area for the spectra depicted in Figure 5.

**Figure A1:**
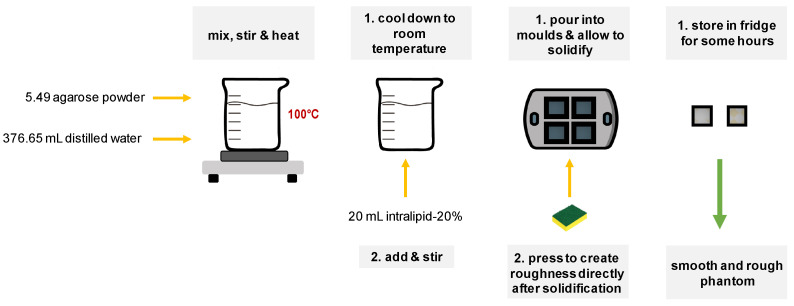
Flowchart illustrating the preparation process for optical phantoms with a smooth and rough surface. Both phantoms have identical chemical compositions, consisting of a mixture of agarose, water, and intralipid. After solidification, the rough side of a cleaning scouring pad is pressed onto one phantom’s surface. This produces a phantom with a rough surface.

We made two homogeneous optical phantoms that were identical in terms of chemical composition: one with a smooth surface and the other with a rough surface. The phantoms consisted of a mix of Intralipid-20% (Fresenius Kabi, Bad Homburg, Germany) and agarose. The latter was incorporated to solidify the phantoms, enabling us to create surface roughness. We mixed 5.49 g of agarose powder (A0169, Sigma-Aldrich, Dorset, UK), 376.65 mL of distilled water and 20 mL of Intralipid-20%. This dilution results in a reduced scattering coefficient varying from 0.8 to 1.65 mm^−1^ for wavelengths between 500 to 900 nm [42]. We placed a glass beaker with agarose and water on a magnetic stirrer hot plate (Thermo Fisher Scientific, Shanghai, China). The agarose solution was magnetically stirred at a speed of 300 rpm and simultaneously heated to a temperature of 100 °C for a couple of minutes until the agarose solution became transparent. After it cooled down to room temperature, the Intralipid-20% was added. Then, the mixture was poured into two identical molds. Immediately after the phantoms solidified, we took one phantom and pressed the rough side of a cleaning scouring pad (medium grit size of roughly 0.10 mm) onto the phantom’s surface. This generated surface roughness in one of the two phantoms. After both phantoms were solidified, they were covered with cling film (to minimize evaporation and contamination) and placed in the fridge for a couple of hours. A summary of the phantom preparation process is illustrated in Figure A1 of the Appendix A. Both phantoms (3 cm high) were cut into a square of ±4 × 4 cm^2^ as depicted in Figure 4. This figure shows pseudo-RGB images of the phantoms generated from the hypercubes and centrally cropped to 550 × 300 pixels. Wavelength bands at 900, 752, and 602 nm were assigned to the red, green, and blue channels, respectively. To analyze the phantoms, we used the acquired hypercubes to select a region of interest (ROI) on each phantom to examine the influence of the phantom’s surface roughness on the measured reflectance.

### 2.6. Excised Human Breast Tissue

To investigate our approach in a more clinical context, we performed measurements on excised breast tissue from a female patient who underwent primary breast-conserving surgery at the Netherlands Cancer Institute/Antoni van Leeuwenhoek Hospital (Amsterdam, The Netherlands). The measurements were performed immediately after surgery on the excised breast tissue. This ex vivo study was approved by the Institutional Review Board of the Netherlands Cancer Institute/Antoni van Leeuwenhoek Hospital (protocol number CFMPB545) and was performed in compliance with the Declaration of Helsinki. According to the Dutch Medical Research Involving Human Subjects Act (WMO), obtaining written informed consent from the patient was not mandated in this specific case.

To investigate our approach, we identified three ROIs within the sample on the hypercubes, which were centrally cropped to a size of 550 × 500 pixels. These regions of 3 by 3 pixels corresponded to various tissue types, including malignant invasive carcinoma, adipose tissue, and connective tissue, as indicated by histopathological ground-truth labels. Since each ROI was intentionally selected to encompass an area of 0.48 by 0.48 mm comprising identical tissue type, it is expected that there is almost no variation of the volume reflectance within the ROI.

The ground-truth labels were derived by marking specific regions of the hyperspectral image with black ink, which were then correlated with H&E stained images obtained through histopathology. This correlation enabled the identification of regions corresponding to the gold standard in pathology. To obtain ground-truth labels, the ink marks on the H&E images were traced back, outlining a 2 mm area directly beneath the visible ink mark [31,32,43]. Subsequently, a pathologist annotated this delineated region. For a more comprehensive understanding of the acquisition process involving hyperspectral images containing ink marks and the annotation of delineated regions, please refer to our previous paper [32], where we elaborate on the methodology.

### 2.7. Evaluation Performance

To measure the performance of our fit, we used the root mean square error (*RMSE*) as a statistical metric. The *RMSE* represents the distance between the measured data of the phantom with a smooth surface and the predicted data of the phantom with a rough surface and can be calculated by

(7) $$RMSE=\sqrt{\frac{\sum_{k=1}^{n}({\hat{R}}_{k}-R_{k}{)}^{2}}{n}}$$

where ${\hat{R}}_{k}$ is the predicted reflectance for wavelength *k* in the phantom with a rough surface, $R_{k}$ the measured reflectance averaged over all illumination directions for wavelength *k* in the phantom with a smooth surface and *n*, the total length of the wavelength range. Generally, a lower *RMSE* indicates a better correlation between the predicted and the measured data.

## 3. Results

**Figure 5:**
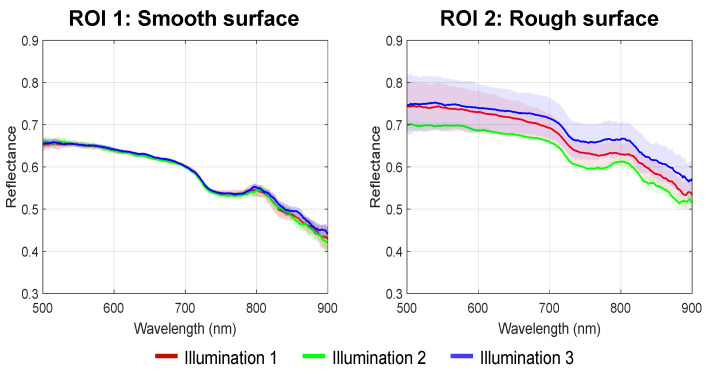
Average reflectance spectra of the tissue-mimicking phantoms with different surface structure. The spectra with shaded STD correspond to ROI 1 (**left**) and ROI 2 (**right**) of Figure 4 and are depicted for each illumination direction separately. The spectra of ROI 1 show a similar reflectance due to the phantom’s smooth surface, while the spectra of ROI 2 differ in reflectance due to the additional surface reflectance as a result of the phantom’s surface roughness.

**Figure 6:**
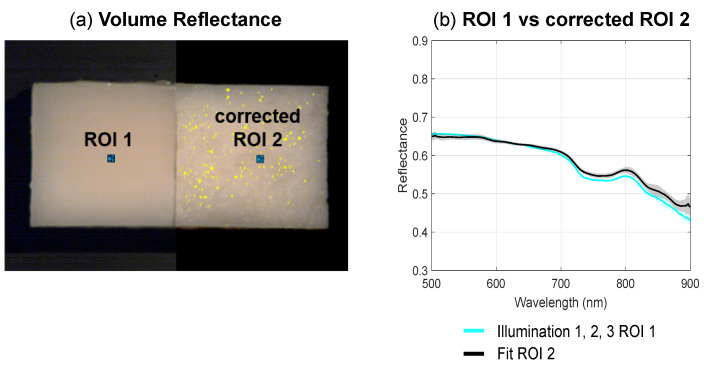
Tissue-mimicking phantoms after separating the volume and surface reflectance in the right phantom. (**a**) After separation, the right phantom with a rough surface more closely resembles the left phantom with a smooth surface. The yellow spots indicate saturated pixels as a result of specular reflectance. These pixels have been removed from the analysis. (**b**) Comparison of the average volume reflectance spectra for ROI 1 taken across all illumination directions (cyan), with the average volume reflectance spectra for the corrected ROI 2 (black), accompanied by their respective shaded STD. Without surface reflectance, the spectra of the corrected ROI 2 are similar to the spectra of ROI 1.

In Figure 4, pseudo-RGB images are shown that were obtained from the hypercubes for each illumination direction. The left phantom has a smooth surface, whereas the right phantom has a rough surface. These images clearly show that, as intended, the phantom with a smooth surface maintains a smooth reflectance across the entire sample and for each illumination direction. Conversely, the reflectance of the phantom with a rough surface varies significantly across the sample and, in addition, changes depending on the direction of illumination. To compare the spectral data of the phantoms, a region of 25 pixels was selected in the center of the left (ROI 1) and right phantom (ROI 2). For both regions, the corresponding average reflectance spectra with their shaded STD are shown in Figure 5. It can be noticed that the spectra in ROI 1 are close to identical for all illumination directions (i.e., red, green, and blue lines), while the spectra in ROI 2 show larger variations between illumination directions. Figure 6a represents the result of separating the volume and surface reflectance in the phantom with the rough surface. This image was overlaid on the original image before the separation, resulting in two phantoms with only volume reflectance. To compare the volume reflections of these phantoms, the spectra of ROI 1 were averaged over all directions (cyan line) and shown along with the average spectra of the corrected ROI 2 without surface reflectance (black line). From this graph (Figure 6b), a distinct similarity between the reflectance spectra can be observed. This is also indicated by the RMSE, which was 0.015 ± 0.003 (mean ± STD). When comparing the average deviation of the measured reflectance of the spectra in ROI 2 across all three illumination directions to the average deviation of the corrected reflectance of the spectra in ROI 2, the spectral variations have been reduced by 83%.

**Figure 7:**
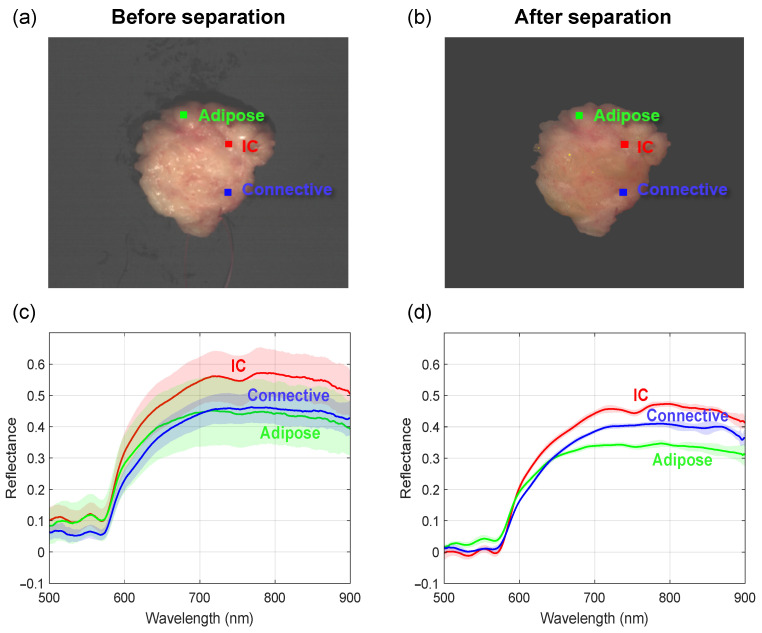
Example of human breast tissue before and after separating the reflections. (**a**,**b**) Pseudo-RGB hyperspectral image of the breast tissue with (left) and without (right) surface reflectance. The image dimensions are 550 × 500 pixels. Three regions of 9 pixels are selected, indicating the locations where adipose, invasive carcinoma, and connective tissue are present. The yellow spots represent saturated pixels due to specular reflectance. These pixels were not used in the analysis. (**c**,**d**) Reflectance spectra averaged over three illumination directions for each tissue type. The shaded area represents the STD. (**c**) Measured spectra before separation of the reflections, revealing a distinct overlap between various tissue types due to variations in surface reflectance. (**d**) Volume reflectance spectra after separation illustrating a clear spectral differentiation among various tissue types.

Figure 7a and Figure 7b show the excised human breast tissue before and after separating the reflections, respectively. Three regions of interest were analyzed that each consisted of 9 pixels. Each of these regions indicates a different tissue type as confirmed by histopathology, i.e., adipose (fat), invasive carcinoma (tumorous tissue), and connective tissue. Although the volume reflectance per tissue region is expected to be similar, the corresponding average spectra do show large STDs (shaded areas) before the separation (Figure 7c). As a result of these large variations, there is also much spectral overlap between the spectra from the three tissue regions, showing no clear differences in spectral shape. In Figure 7b, the pseudo-RGB image shows the breast tissue following the separation of the reflections. The surface reflectance has been removed, and thus, the image of the breast tissue only contains the calculated volume reflectance. It can be observed that the edges of the tissue appear slightly darker. After separating volume and surface reflectance, the average volume reflectance spectra with STD are depicted in Figure 7d. In this context, the spectra exhibit noticeable differences among tissue regions characterized by minimal STDs.

## 4. Discussion

The research conducted by Kho et al. [27], which revealed substantial pixel-to-pixel variability within spectra, motivated us to investigate its underlying cause. Building upon their findings, we hypothesized that this variability, distinct from bulk tissue characteristics, could arise from significant variations in surface reflectance across pixels. This hypothesis was successfully confirmed in optical phantoms and an excised human breast tissue sample.

Our experiment on optical phantoms (Figure 4, Figure 5 and Figure 6) demonstrated the impact of surface irregularities on reflectance intensity in hyperspectral images and spectra. The phantom with a rough surface exhibited distinct fluctuations in reflectance, resulting in larger standard deviations in spectra compared to the phantom with a smooth surface. Applying our approach reduced spectral variations by 83% in the phantom with a rough surface, indicating its effectiveness. Additionally, with an RMSE of only 0.015 ± 0.003 between the volume reflectance spectra of the identified regions on the phantoms with a smooth and rough surface after separation (Figure 6), our approach demonstrated its ability to determine volume reflectance accurately.

Studying excised human breast tissue after surgery posed significant challenges in data analysis. Surface reflectance from the tissue complicated the differentiation of tissue regions such as invasive carcinoma, connective tissue, and adipose tissue (Figure 7). Despite varying tissue compositions, surface reflectance caused spectral variations, resulting in large variability within homogeneous tissue types and overlap between different tissue regions. Our method successfully addressed these challenges, enabling precise differentiation of tissue regions based on their spectral characteristics.

**Figure 8:**
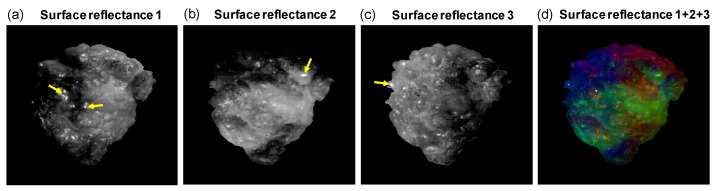
Surface reflectance image of human breast tissue. (**a**–**c**) Estimated surface reflectance from three different illumination directions highlighting the tissue’s surface structure. The yellow spots indicated by arrows represent saturated pixels as a result of specular reflectance. (**d**) Pseudo-RGB image of the tissue’s surface reflectance from the three illumination directions combined. All images have dimensions of 350 × 350 pixels.

Considering surface reflectance, our approach allowed for the calculation of the proportionality constant ε for each pixel per illumination direction (Equation (4)). These results, depicted in Figure 8a–c, were combined into a single pseudo-RGB image (Figure 8d). Upon examining this image, we noted several distinct observations: first, shadows or surface inclination gradients were visible as colored areas, appearing more blurred than as discrete points, indicating a systematic reduction in one or a combination of reflections. Second, surface roughness was demonstrated by the presence of bright-colored or white pixels, with each illumination direction yielding a unique reflectance and, consequently, a distinct ε value. Third, in larger regions with surface roughness, numerous pixels displayed varied and intense colors, lacking uniformity and resembling a sort of color noise. Additionally, regions of high roughness exhibited increased intensity, while regions of low roughness showed lower intensity levels. Moreover, it was noted from this image that our approach was less effective at the sample edges, as indicated by the blurred colored areas. Furthermore, the image revealed variations in surface roughness across the sample. Thus, it appears that surface roughness can be effectively highlighted using our approach. Besides providing a better estimation of volume reflectance, our method also enables visualization of surface roughness, which could provide additional diagnostic value.

Multiple methods currently exist to address surface reflectance, including the use of polarization filters [44,45] and the application of preprocessing techniques [27,46,47,48,49]. Polarization filters have been used [44,45] to eliminate surface reflectance. One disadvantage of polarization filters is that they not only remove surface reflectance but also reduce the detected intensity of the volume reflectance by at least 50%. Moreover, the method has limited functionality when using larger viewing and illumination angles. Furthermore, multiplicative scatter correction (MSC) and standard normal variate (SNV) are two preprocessing techniques that are often used as an approach to minimize the influence of surface reflectance [46]. MSC normalizes spectra to a reference spectrum (e.g., the mean spectrum), while SNV corrects the spectra to a mean of zero and a standard deviation of one. While these preprocessing techniques eliminate the baseline offset from the spectra, they also reduce the variability in the baseline. Consequently, this results in a loss of amplitude and scattering information, which may reduce the diagnostic accuracy. In contrast to these methods, our approach fully preserves volume reflectance and explicitly identifies the information within surface reflectance rather than discarding it. If surface reflectance contains any relevant information for the application, our approach can effectively extract it. Therefore, it presents opportunities for enhancing the analysis and performance of hyperspectral imaging. Nevertheless, further research is necessary to determine whether the separation of surface reflectance from volume reflectance would indeed yield additional improvements for the sample under investigation.

For a clinical application, it could be interesting to explore if the surface reflectance information could aid during complex surgical procedures. In these surgical settings, achieving uniform tissue illumination is a critical objective to facilitate effective hyperspectral image analysis. However, this goal is often obstructed by issues such as shadow areas and surface reflectance caused by the tissue’s morphology [43,50]. By effectively separating the surface reflectance and volume reflectance, our approach may offer the potential for more precise tissue analysis during surgery.

Another potential clinical application is ocular tissue imaging, particularly for investigating and detecting retinal diseases such as age-related macular degeneration, a leading cause of vision loss [51,52,53,54]. Since HSI can capture spectral information to possibly detect changes in the retinal pigment epithelium associated with the progression of this condition, our approach may aid in providing more precise spectral differentiation for assessing retinal health and (early-stage) disease. Additionally, it may enhance the visualization of retinal structures. However, further research is needed to investigate these potential benefits.

Since our approach involves capturing images from multiple illumination directions, it is important to consider that implementing this method requires a slightly larger setup (due to the illumination ring) and additional time for data acquisition. While using a push-broom camera typically takes no more than two minutes in total for three directions, this duration should be taken into account. If faster acquisition is needed, our approach can be easily adapted for use with other hyperspectral imaging configurations, such as snapshot cameras. For in vivo settings, snapshot cameras offer advantages by capturing the entire scene in one shot, both spectrally and spatially [55]. This can significantly reduce measurement time and minimize motion artifacts [56]. Additionally, the compact size of snapshot cameras simplifies alignment with the illumination ring and enables closer mounting near the patient compared to push-broom cameras. Furthermore, illumination rings can be customized for various lighting conditions, and switching to a smaller programmable LED version could further enhance fit and efficiency, particularly when adjusting illumination directions.

When measuring samples with surfaces that have curved edges, the varying angle of light incidence along these edges significantly impacts the amount of light that can contribute to volume reflectance. This variation poses a challenge in maintaining consistent zenith angles. As a result, our method may not be effective for regions near the sample edges where constant volume reflectance cannot be achieved. Nevertheless, our method demonstrates effectiveness away from the edges, indicating its usefulness for accurately capturing volume reflectance measurements in those regions.

In summary, we have presented our approach for addressing non-bulk tissue-related spectral variations in hyperspectral images. We have validated its effectiveness through successful demonstrations on both tissue-mimicking phantoms and excised human breast tissue. To the best of our knowledge, this is the first study that conserves the potential information contained within surface reflectance. This information holds promise for diagnostic applications and could play an important role in enhancing sample analysis and evaluation. It is worth mentioning that the integration of the introduced technique into clinical practice necessitates rigorous standardization and validation to ensure consistent and accurate results across diverse medical applications. The current landscape of HSI technology reveals several promising advancements aimed at addressing these critical requirements [57]. While we have elaborated on the clinical application of our approach, it is worth noting that this approach necessitates only minor adjustments to the illumination setup and data analysis. As such, it can be applied across a wide spectrum of fields where hyperspectral imaging is used.

## 5. Conclusions

In this study, we have investigated a novel approach to address non-bulk tissue-related spectral variations in medical hyperspectral imaging. This was based on the hypothesis that spectral variability originated from significant variations in surface reflectance across pixels. Our findings demonstrated an 83% reduction in spectral variability using our approach on optical phantoms. Thus, we effectively validated our approach on optical phantoms and demonstrated its functionality on excised human breast tissue. This approach is a significant advantage when dealing with samples of unknown refractive index spectra. With the capability to conserve surface reflectance information, our approach has the potential to uncover additional insights into sample properties, holding promise for diagnostic applications and enhancing sample analysis and evaluation. However, further research is required to fully understand its potential in improving the characterization of the sample under investigation. Future experiments in clinical diagnostics could give a comprehensive demonstration of the proposed method. Given its minimal requirements for setup adjustments and data analysis, our approach can be easily adapted and applied across a wide spectrum of fields where hyperspectral imaging is used.

## Data Availability

The data presented in this study are available on request from the corresponding author. The data are not publicly available.

## References

[1] Gowen A.A., O’Donnell C.P., Cullen P.J., Downey G., Frias J.M. (2007). Hyperspectral imaging–an emerging process analytical tool for food quality and safety control. Trends Food Sci. Technol..

[2] Edelman G.J., Gaston E., van Leeuwen T.G., Cullen P., Aalders M.C. (2012). Hyperspectral imaging for non-contact analysis of forensic traces. Forensic Sci. Int..

[3] Li Q., He X., Wang Y., Liu H., Xu D., Guo F. (2013). Review of spectral imaging technology in biomedical engineering: Achievements and challenges. J. Biomed. Opt..

[4] Lu G., Fei B. (2014). Medical hyperspectral imaging: A review. J. Biomed. Opt..

[5] Adam E., Mutanga O., Rugege D. (2010). Multispectral and hyperspectral remote sensing for identification and mapping of wetland vegetation: A review. Wetl. Ecol. Manag..

[6] Van der Meer F.D., van der Werff H.M., van Ruitenbeek F.J., Hecker C.A., Bakker W.H., Noomen M.F., van der Meijde M., Carranza E.J.M., de Smeth J.B., Woldai T. (2012). Multi-and hyperspectral geologic remote sensing: A review. Int. J. Appl. Earth Obs. Geoinf..

[7] Dale L.M., Thewis A., Boudry C., Rotar I., Dardenne P., Baeten V., Pierna J.A.F. (2013). Hyperspectral imaging applications in agriculture and agro-food product quality and safety control: A review. Appl. Spectrosc. Rev..

[8] Mahesh S., Jayas D., Paliwal J., White N. (2015). Hyperspectral imaging to classify and monitor quality of agricultural materials. J. Stored Prod. Res..

[9] Sun D.W. (2010). Hyperspectral Imaging for Food Quality Analysis and Control.

[10] Pu Y.Y., Feng Y.Z., Sun D.W. (2015). Recent progress of hyperspectral imaging on quality and safety inspection of fruits and vegetables: A review. Compr. Rev. Food Sci. Food Saf..

[11] Feng Y.Z., Sun D.W. (2012). Application of hyperspectral imaging in food safety inspection and control: A review. Crit. Rev. Food Sci. Nutr..

[12] Schuler R.L., Kish P.E., Plese C.A. (2012). Preliminary observations on the ability of hyperspectral imaging to provide detection and visualization of bloodstain patterns on black fabrics. J. Forensic Sci..

[13] Liang H. (2012). Advances in multispectral and hyperspectral imaging for archaeology and art conservation. Appl. Phys. A.

[14] Doneus M., Verhoeven G., Atzberger C., Wess M., Ruš M. (2014). New ways to extract archaeological information from hyperspectral pixels. J. Archaeol. Sci..

[15] Yuen P.W., Richardson M. (2010). An introduction to hyperspectral imaging and its application for security, surveillance and target acquisition. Imaging Sci. J..

[16] Briottet X., Boucher Y., Dimmeler A., Malaplate A., Cini A., Diani M., Bekman H., Schwering P., Skauli T., Kasen I. (2006). Military applications of hyperspectral imagery. Proceedings of the Targets and Backgrounds XII: Characterization and Representation.

[17] Brown A.J., Walter M.R., Cudahy T. (2005). Hyperspectral imaging spectroscopy of a Mars analogue environment at the North Pole Dome, Pilbara Craton, Western Australia. Aust. J. Earth Sci..

[18] Stuffler T., Förster K., Hofer S., Leipold M., Sang B., Kaufmann H., Penné B., Mueller A., Chlebek C. (2009). Hyperspectral imaging—An advanced instrument concept for the EnMAP mission (Environmental Mapping and Analysis Programme). Acta Astronaut..

[19] Schultz R.A., Nielsen T., Zavaleta J.R., Ruch R., Wyatt R., Garner H.R. (2001). Hyperspectral imaging: A novel approach for microscopic analysis. Cytometry.

[20] Akbari H., Kosugi Y., Kojima K., Tanaka N. Blood vessel detection and artery-vein differentiation using hyperspectral imaging. Proceedings of the 2009 Annual International Conference of the IEEE Engineering in Medicine and Biology Society.

[21] Akbari H., Kosugi Y., Kojima K., Tanaka N. (2010). Detection and analysis of the intestinal ischemia using visible and invisible hyperspectral imaging. IEEE Trans. Biomed. Eng..

[22] Sersa G., Simoncic U., Milanic M. (2022). Imaging perfusion changes in oncological clinical applications by hyperspectral imaging: A literature review. Radiol. Oncol..

[23] Hren R., Stergar J., Simončič U., Serša G., Milanič M. (2024). Assessing Perfusion Changes in Clinical Oncology Applications Using Hyperspectral Imaging. Proceedings of the European Medical and Biological Engineering Conference.

[24] Khoobehi B., Beach J.M., Kawano H. (2004). Hyperspectral imaging for measurement of oxygen saturation in the optic nerve head. Investig. Ophthalmol. Vis. Sci..

[25] Mordant D., Al-Abboud I., Muyo G., Gorman A., Harvey A., McNaught A. (2014). Oxygen saturation measurements of the retinal vasculature in treated asymmetrical primary open-angle glaucoma using hyperspectral imaging. Eye.

[26] Johnson W.R., Wilson D.W., Fink W., Humayun M., Bearman G. (2007). Snapshot hyperspectral imaging in ophthalmology. J. Biomed. Opt..

[27] Kho E., de Boer L.L., van de Vijver K.K., van Duijnhoven F., Vrancken Peeters M.J.T., Sterenborg H.J., Ruers T.J. (2019). Hyperspectral imaging for resection margin assessment during cancer surgery. Clin. Cancer Res..

[28] Kho E., Dashtbozorg B., de Boer L.L., van de Vijver K.K., Sterenborg H.J., Ruers T.J. (2019). Broadband hyperspectral imaging for breast tumor detection using spectral and spatial information. Biomed. Opt. Express.

[29] Kho E., de Boer L.L., Post A.L., Van de Vijver K.K., Jóźwiak K., Sterenborg H.J., Ruers T.J. (2019). Imaging depth variations in hyperspectral imaging: Development of a method to detect tumor up to the required tumor-free margin width. J. Biophotonics.

[30] Baltussen E.J., Kok E.N., Brouwer de Koning S.G., Sanders J., Aalbers A.G., Kok N.F., Beets G.L., Flohil C.C., Bruin S.C., Kuhlmann K.F. (2019). Hyperspectral imaging for tissue classification, a way toward smart laparoscopic colorectal surgery. J. Biomed. Opt..

[31] Jong L.J.S., de Kruif N., Geldof F., Veluponnar D., Sanders J., Peeters M.J.T.V., van Duijnhoven F., Sterenborg H.J., Dashtbozorg B., Ruers T.J. (2022). Discriminating healthy from tumor tissue in breast lumpectomy specimens using deep learning-based hyperspectral imaging. Biomed. Opt. Express.

[32] Jong L.J.S., Post A.L., Veluponnar D., Geldof F., Sterenborg H.J., Ruers T.J., Dashtbozorg B. (2023). Tissue Classification of Breast Cancer by Hyperspectral Unmixing. Cancers.

[33] Witteveen M., Sterenborg H.J., van Leeuwen T.G., Aalders M.C., Ruers T.J., Post A.L. (2022). Comparison of preprocessing techniques to reduce nontissue-related variations in hyperspectral reflectance imaging. J. Biomed. Opt..

[34] Li B., Beveridge P., O’Hare W.T., Islam M. (2013). The age estimation of blood stains up to 30 days old using visible wavelength hyperspectral image analysis and linear discriminant analysis. Sci. Justice.

[35] Collins T., Maktabi M., Barberio M., Bencteux V., Jansen-Winkeln B., Chalopin C., Marescaux J., Hostettler A., Diana M., Gockel I. (2021). Automatic recognition of colon and esophagogastric cancer with machine learning and hyperspectral imaging. Diagnostics.

[36] Maktabi M., Köhler H., Ivanova M., Jansen-Winkeln B., Takoh J., Niebisch S., Rabe S.M., Neumuth T., Gockel I., Chalopin C. (2019). Tissue classification of oncologic esophageal resectates based on hyperspectral data. Int. J. Comput. Assist. Radiol. Surg..

[37] Malegori C., Alladio E., Oliveri P., Manis C., Vincenti M., Garofano P., Barni F., Berti A. (2020). Identification of invisible biological traces in forensic evidences by hyperspectral NIR imaging combined with chemometrics. Talanta.

[38] Peñaranda F., Naranjo V., Lloyd G.R., Kastl L., Kemper B., Schnekenburger J., Nallala J., Stone N. (2018). Discrimination of skin cancer cells using Fourier transform infrared spectroscopy. Comput. Biol. Med..

[39] Pardo A., Real E., Krishnaswamy V., López-Higuera J.M., Pogue B.W., Conde O.M. (2016). Directional kernel density estimation for classification of breast tissue spectra. IEEE Trans. Med. Imaging.

[40] Welch A.J., van Gemert M.J. (2011). Optical-Thermal Response of Laser-Irradiated Tissue.

[41] Flock S.T., Patterson M.S., Wilson B.C., Wyman D.R. (1989). Monte Carlo modeling of light propagation in highly scattering tissues. I. Model predictions and comparison with diffusion theory. IEEE Trans. Biomed. Eng..

[42] Cubeddu R., Pifferi A., Taroni P., Torricelli A., Valentini G. (1997). A solid tissue phantom for photon migration studies. Phys. Med. Biol..

[43] Kho E., Dashtbozorg B., Sanders J., Vrancken Peeters M.J.T., van Duijnhoven F., Sterenborg H.J., Ruers T.J. (2021). Feasibility of ex vivo margin assessment with hyperspectral imaging during breast-conserving surgery: From imaging tissue slices to imaging lumpectomy specimen. Appl. Sci..

[44] Keresztes J.C., Koshel R.J., Chipman R., Stover J.C., Saeys W. (2015). A cross-polarized freeform illumination design for glare reduction in fruit quality inspection. Proceedings of the Optical Systems Design 2015: Illumination Optics IV.

[45] Nguyen-Do-Trong N., Keresztes J.C., De Ketelaere B., Saeys W. (2016). Cross-polarised VNIR hyperspectral reflectance imaging system for agrifood products. Biosyst. Eng..

[46] Rinnan Å., Van Den Berg F., Engelsen S.B. (2009). Review of the most common pre-processing techniques for near-infrared spectra. Trac Trends Anal. Chem..

[47] Barnes R., Dhanoa M.S., Lister S.J. (1989). Standard normal variate transformation and de-trending of near-infrared diffuse reflectance spectra. Appl. Spectrosc..

[48] Keresztes J.C., Goodarzi M., Saeys W. (2016). Real-time pixel based early apple bruise detection using short wave infrared hyperspectral imaging in combination with calibration and glare correction techniques. Food Control.

[49] Claridge E., Hidović-Rowe D. (2013). Model based inversion for deriving maps of histological parameters characteristic of cancer from ex-vivo multispectral images of the colon. IEEE Trans. Med. Imaging.

[50] Lai M., van der Stel S.D., Groen H.C., van Gastel M., Kuhlmann K.F., Ruers T.J., Hendriks B.H. (2022). Imaging PPG for in vivo human tissue perfusion assessment during surgery. J. Imaging.

[51] Meleppat R.K., Ronning K.E., Karlen S.J., Kothandath K.K., Burns M.E., Pugh E.N., Zawadzki R.J. (2020). In situ morphologic and spectral characterization of retinal pigment epithelium organelles in mice using multicolor confocal fluorescence imaging. Investig. Ophthalmol. Vis. Sci..

[52] Ami T.B., Tong Y., Bhuiyan A., Huisingh C., Ablonczy Z., Ach T., Curcio C.A., Smith R.T. (2016). Spatial and spectral characterization of human retinal pigment epithelium fluorophore families by ex vivo hyperspectral autofluorescence imaging. Transl. Vis. Sci. Technol..

[53] Pascolini D., Mariotti S.P. (2012). Global estimates of visual impairment: 2010. Br. J. Ophthalmol..

[54] Wong W.L., Su X., Li X., Cheung C.M.G., Klein R., Cheng C.Y., Wong T.Y. (2014). Global prevalence of age-related macular degeneration and disease burden projection for 2020 and 2040: A systematic review and meta-analysis. Lancet Glob. Health.

[55] Halicek M., Fabelo H., Ortega S., Callico G.M., Fei B. (2019). In-vivo and ex-vivo tissue analysis through hyperspectral imaging techniques: Revealing the invisible features of cancer. Cancers.

[56] Jong L.J.S., Appelman J.G., Sterenborg H.J., Ruers T.J., Dashtbozorg B. (2024). Spatial and Spectral Reconstruction of Breast Lumpectomy Hyperspectral Images. Sensors.

[57] Stergar J., Hren R., Milanič M. (2022). Design and validation of a custom-made laboratory hyperspectral imaging system for biomedical applications using a broadband LED light source. Sensors.

